# Invasive Fusariosis in Pediatric Hematology/Oncology and Stem Cell Transplant Patients: A Report from the Israeli Society of Pediatric Hematology-Oncology

**DOI:** 10.3390/jof8040387

**Published:** 2022-04-11

**Authors:** Marganit Benish, Sarah Elitzur, Nira Arad-Cohen, Assaf Arie Barg, Miriam Ben-Harosh, Bella Bielorai, Salvador Fischer, Gil Gilad, Itzhak Levy, Hila Rosenfeld-Keidar, Yael Shachor-Meyouhas, Galia Soen-Grisaru, Sigal Weinreb, Ronit Nirel, Ronit Elhasid

**Affiliations:** 1 Department of Pediatric Hemato-Oncology, Sourasky Medical Center, Tel Aviv 6423906, Israel; marganitbe@tlvmc.gov.il (M.B.); hilark@tlvmc.gov.il (H.R.-K.); ronite@tlvmc.gov.il (R.E.); 2 Sackler Faculty of Medicine, Tel Aviv University, Tel Aviv 6997801, Israel; assaf.barg@sheba.health.gov.il (A.A.B.); bella.bielorai@sheba.health.gov.il (B.B.); salvadorf@clalit.org.il (S.F.); gilgi@clalit.org.il (G.G.); itzhakl@clalit.org.il (I.L.); galiag@tlvmc.gov.il (G.S.-G.); 3 The Rina Zaizov Division of Pediatric Hematology-Oncology, Schneider Children’s Medical Center, Petah Tikva 4920235, Israel; 4 Pediatric Hematology-Oncology Department, Ruth Rappaport Children’s Hospital, Rambam Health Care Campus, Haifa 3109601, Israel; n_arad-cohen@rambam.health.gov.il; 5 Rappaport Faculty of Medicine, Technion-Israel Institute of Technology, Haifa 3200003, Israel; y_shahor@rambam.health.gov.il; 6 Division of Pediatric Hematology, Oncology and BMT, The Edmond and Lily Safra Children’s Hospital, Sheba Medical Center, Ramat Gan 5262161, Israel; 7 Department of Pediatric Hematology-Oncology, Soroka Medical Center, Ben Gurion University, Beer Sheva 8489501, Israel; miribh@clalit.org.il; 8 Pediatric Infectious Disease Unit, Schneider Children’s Medical Center, Petah Tikva 4920235, Israel; 9 Pediatric Infectious Disease Unit, Ruth Rappaport Children’s Hospital, Rambam Health Care Campus, Haifa 3109601, Israel; 10 Pediatric Infectious Disease Unit, Sourasky Medical Center, Tel Aviv 6423906, Israel; 11 Pediatric Hematology-Oncology, Hadassah Hebrew University Medical Center, Jerusalem 9112000, Israel; sigalv@hadassah.org.il; 12 Department of Statistics and Data Science, Hebrew University, Jerusalem 9190501, Israel; nirelr@mail.huji.ac.il

**Keywords:** children, cancer, immunocompromised, invasive fungal infections, fusarium, pediatric hematology oncology, leukemia, stem cell transplantation

## Abstract

Invasive *Fusarium* species infections in immunocompromised patients occur predominantly in those with hematological malignancies. Survival rates of 20–40% were reported in adults, but data in children are limited. Our retrospective, nationwide multicenter study of invasive fusariosis in pediatric hematology/oncology and stem cell transplant (SCT) patients identified twenty-two cases. Underlying conditions included hematological malignancies (*n* = 16; 73%), solid tumors (*n* = 2), and non-malignant hematological conditions (*n* = 4). Nineteen patients (86%) were neutropenic, nine (41%) were SCT recipients, and seven (32%) received corticosteroids. Sixteen patients (73%) had disseminated fusariosis, five had local infection, and one had isolated fungemia. Fifteen patients (68%) had skin involvement and eight (36%) had a bloodstream infection. Four patients (18%) presented with osteoarticular involvement and four with pulmonary involvement. Nineteen patients (86%) received combination antifungal therapy upfront and three (14%) received single-agent treatment. Ninety-day probability of survival was 77%: four of the five deaths were attributed to fusariosis, all in patients with relapsed/refractory acute leukemias. Ninety-day probability of survival for patients with relapsed/refractory underlying malignancy was 33% vs. 94% in others (*p* < 0.001). Survival rates in this largest pediatric population-based study were strikingly higher than those reported in adults, demonstrating that invasive fusariosis is a life-threatening but salvageable condition in immunosuppressed children.

## 1. Introduction

Invasive fungal infections are an important cause of morbidity and mortality in immunocompromised patients. An increasing prevalence of invasive mold infections has been reported in patients with hematological malignancies in recent decades [1]. *Aspergillus* species are the major cause of invasive mold infections, but other rare opportunistic non-*Aspergillus* molds are increasingly encountered, including *Fusarium*, Mucorales, and *Scedosporium* species [2]. *Fusarium* species are common environmental fungi capable of causing infections in both animals and plants. *Fusarium* species infections in immunocompetent individuals are generally noninvasive, and frequently manifest as onychomycosis or keratitis. Invasive fusariosis may occur in immunocompromised hosts, often with disseminated disease and poor outcomes [3], and mortality rates as high as 60–80% have been reported in adult studies [4]. Poor outcomes have been attributed, in part, to an intrinsic resistance to multiple antifungal agents, as demonstrated by in vitro antifungal susceptibility tests [5]. The incidence of invasive fusariosis varies depending on the underlying disease and geographical region [4].

There is a paucity of data from controlled clinical trials to guide the management of rare invasive fungal infections, and data on these fungal infections in children are even rarer [6,7,8,9,10], with management guidelines frequently derived from adult studies. Data on *Fusarium* infections in pediatric immunosuppressed patients are especially scarce, and they are primarily limited to case reports and small series [11,12,13,14,15,16,17,18,19,20,21]. We designed this multicenter population-based study to investigate the characteristics, treatment, and outcome of invasive fusariosis in pediatric hematology/oncology and stem cell transplant (SCT) patients.

## 2. Materials and Methods

### 2.1. Study Design

We conducted a retrospective search for cases of invasive fusariosis in all six pediatric hematology–oncology departments in Israel. The medical records, microbiology databases, and pathology information systems in each center were searched for all cases of invasive fusariosis that occurred between 1 July 2005 and 1 May 2021 involving patients aged 0–18 years. Fusariosis was categorized as proven or probable based upon the definitions of the European Organization for Research and Treatment of Cancer/Mycoses Study Group (EORTC/MSG) for invasive fungal disease [22]. Cases categorized as possible were excluded. Fusariosis was classified as localized disease, disseminated disease, or fungemia. Disseminated fusariosis was defined as the involvement of two or more non-contiguous sites. Cases of fungemia were not defined as disseminated disease unless another organ was involved.

Patients diagnosed with fusariosis were evaluated by computed tomography (CT) scans of sinuses, chest, and upper abdomen. Corticosteroid use was considered significant if 2 mg/kg prednisolone or equivalent was administered for at least 10 days during the 60 days prior to being diagnosed with invasive fusariosis. Neutropenia was defined as a neutrophil count of ≤500 cells/µL. The primary outcome of the study was the 90-day mortality from the day of *Fusarium* infection diagnosis. Death attributed to fusariosis was defined as any death occurring during active fusarium infection.

### 2.2. Diagnostic Methods

Identification of cultured *Fusarium* species was performed at the various institutional microbiology laboratories using standard phenotypic methods. When possible, fresh specimens were sent to a central reference laboratory for DNA sequencing and analysis. DNA was extracted from clinical samples by means of the QIAamp DNA Mini Kit (Qiagen, Valencia, CA, USA), and amplified with a semi-nested polymerase chain reaction (PCR) assay targeting the 28S rDNA. The PCR products were separated by electrophoresis in ethidium bromide-stained 2% agarose gels, sequenced on a 3130 Genetic Analyzer capillary electrophoresis DNA sequencer (Applied Biosystems, Carlsbad, CA, USA), and analyzed with the Basic Local Alignment Search Tool (BLAST). Testing of antifungal susceptibility was performed locally. The minimal inhibitory concentrations (MIC) of the antifungal agents for each isolate were determined with either broth microdilution or gradient concentration strips (Novamed) on RPMI plates.

### 2.3. Statistical Analysis

Differences in the rates of 90-day mortality among patient subgroups were analyzed with Fisher’s exact test. Overall survival (OS) was calculated as time from the diagnosis of *Fusarium* species infection to death. Survival rates were estimated with the Kaplan–Meier method, and the log-rank test was used to compare survival among groups.

## 3. Results

### 3.1. Patient Characteristics

**Figure 1:**
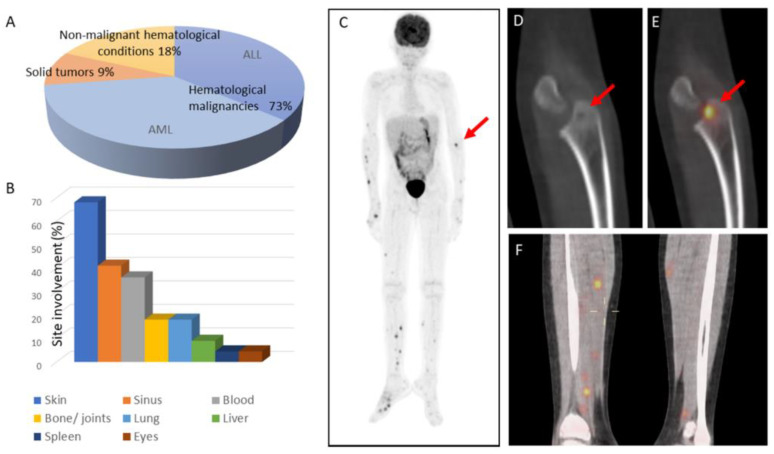
Invasive fusariosis in children: patient characteristics and infection patterns. (**A**) Distribution of underlying conditions in the study cohort. (**B**) Sites of involvement. (**C**) Coronal PET-CT of an 11-year-old boy with AML and disseminated fusariosis, with multiple cutaneous, intramuscular, and skeletal fluorodeoxyglucose (FDG)-avid lesions. A lytic lesion in the left radius is indicated by the arrow. (**D**) CT of a left radial lytic lesion (arrow) (**E**) with pathological FDG uptake (arrow). (**F**) Coronal lower limb PET-CT showing multiple bilateral intramuscular calf lesions with FDG uptake.

Twenty-two children with invasive fusariosis were identified for inclusion in the study. Their demographic characteristics are summarized in Table 1 and in the Supplementary Table S1. The median age of the study group was 10.8 years (range: 0.4–18 years). Sixteen (73%) patients had underlying hematological malignancies: eight had acute myeloid leukemia (AML) and eight had acute lymphoblastic leukemia (ALL, six with relapsed/refractory disease). Two patients had underlying solid tumors, one with a medulloblastoma and the other with a neuroblastoma. Four patients had underlying non-malignant hematological conditions, two with aplastic anemia, one with beta-thalassemia following SCT, and one with neutropenia due to adenosine deaminase deficiency type 2 (ADA2) (Figure 1A). Nine patients (41%) had received chemotherapy before the diagnosis of fusarium infection and nine patients (41%) were SCT recipients (eight received allogeneic SCT and one received autologous SCT). None of the SCT recipients had active graft-versus-host disease (GVHD) at the time of fusariosis. Two patients had received immunotherapy (anti-GD2 antibodies for neuroblastoma and anti-thymocyte globulin with cyclosporin for aplastic anemia), and two patients with non-malignant hematological disorders (aplastic anemia and ADA2 deficiency) had not received any immunosuppressive therapy whatsoever. Only seven patients (32%) received corticosteroid therapy. Nineteen patients (86%) were neutropenic at the time of fusariosis: two had long-lasting, congenital neutropenia (aged 1.1 years and 3.8 years) and seventeen had new-onset neutropenia (median duration: 17 days; range: 6–105 days). The two patients who did not receive any immunosuppressive therapy prior to being diagnosed with invasive fusariosis were those with long-lasting congenital neutropenia. Nineteen patients (86%) had received prophylactic antifungal therapy, mostly fluconazole (*n* = 10) or itraconazole (*n* = 7).

### 3.2. Infection Patterns and Pathogens

**Figure 2:**
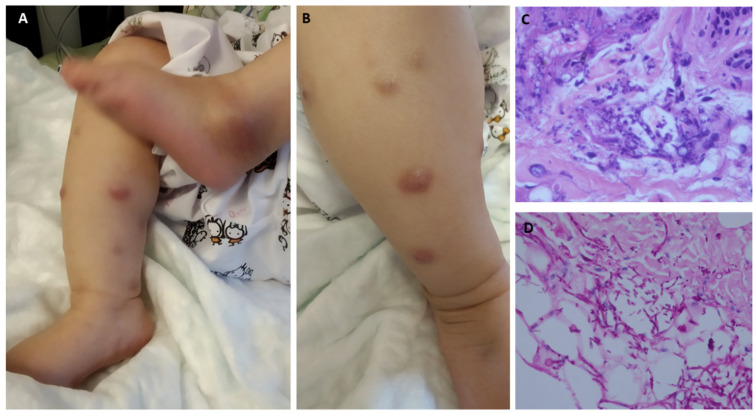
Typical clinical and histopathological features of invasive fusariosis. (**A**,**B**) Erythematous nodular skin lesions and septic arthritis of the left ankle in a 4-month-old girl with AML. (**C**) Biopsy of a cutaneous lesion demonstrating numerous fungal septate hyphae (hematoxylin and eosin, 400×). (**D**) Skin biopsy: periodic acid Schiff stain highlighting fungal elements (200×).

Sixteen patients (73%) presented with disseminated fusariosis. Five of them had a local infection (one localized to the skin and four to the sinuses), and one patient (with medulloblastoma) presented with isolated fungemia. The most common clinical manifestation (15 cases, 68%) was skin involvement comprised of erythematous papular or nodular skin lesions. In total, eight patients (36%) had a bloodstream infection. Notably, four patients (18%) had bone or joint involvement (Figure 1B). Pulmonary involvement was reported in four patients, all with bilateral nodular infiltrates, two with a halo of ground-grass infiltrates, and one with a cavitation. The sites of involvement are depicted in Figure 1 and Figure 2. Nineteen cases were classified as proven according to the EORTC/MSG criteria, and three as probable (Supplementary Table S1). *Fusarium* species were identified in eight cases: *Fusarium solani* (*n* = 5), *Fusarium dimerum* (*n* = 1), *Fusarium proliferatum* (*n* = 1), and *Fusarium moniliforme* (*n* = 1). Antifungal susceptibility testing was performed in eleven cases and the minimal inhibitory concentrations (MIC) were variable, with a wide range of MIC distribution. Specifically, the MIC ranges were 0.5–24 mcg/mL for amphotericin B and 0.47->16 mcg/mL for voriconazole (Table 2).

### 3.3. Treatment

Nineteen patients (86%) received antifungal combination therapy up front: eleven received a liposomal amphotericin B-azole combination (voriconazole (*n* = 6); posaconazole (*n* = 2) and isavuconazole (*n* = 3)), seven received an amphotericin B-voriconazole combination, and one patient was treated with voriconazole and caspofungin. Two patients underwent additional therapy with terbinafine. Three patients (14%) received single-agent treatment: two received voriconazole and one received amphotericin B (Table 1). Overall, twenty-one of the twenty-two study patients (95%) received azole treatment, either voriconazole, posaconazole, or isavuconazole.

Adjunctive treatment modalities included surgical debridement procedures (functional endoscopic sinus surgery, *n* = 7; arthroscopy, *n* = 2; bilateral vitrectomy, *n* = 1), granulocyte colony stimulation factor (G-CSF) administration (*n* = 9), and granulocyte transfusions (*n* = 7).

### 3.4. Outcome

**Figure 3:**
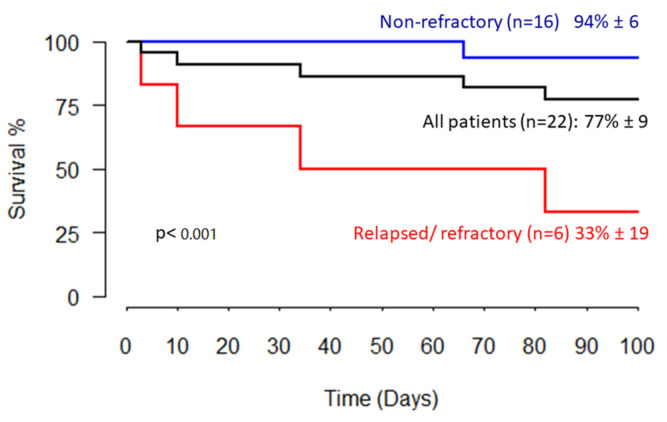
Outcome. Kaplan–Meier 90-day survival curves stratified according to refractory or non-refractory underlying disease.

Five patients (23%) died within 90 days of *Fusarium* species infection diagnosis, four of them (18%) with active fusariosis. All four patients who died with active fusariosis were neutropenic, and each died with relapsed/refractory uncontrolled acute leukemia. The median time to death from the diagnosis of *Fusarium* species infection for these four patients was twenty-two days (range: 3–82 days). The fifth patient, in remission from AML after SCT, had recovered clinically and radiologically from fusariosis but died after 66 days as a result of culture-negative septic shock. The ninety-day probability of survival for the whole cohort was 77%, with 80% of deaths (four of the five) attributable to fusariosis. Neutrophil count recovery was noted in all the patients who survived. The ninety-day probability of survival for patients with relapsed/refractory underlying malignancy was 33%, compared to 94% in those with non-refractory malignancies (*p* < 0.001) (Figure 3). The ninety-day outcome in relation to patient characteristics is summarized in Table 1. Disease status (relapsed/refractory malignancy) was the only variable of statistical significance (*p* = 0.009).

Of the seventeen patients who survived longer than 90 days after the diagnosis of *Fusarium* species infection, four subsequently received intensive chemotherapy, one continued to receive immunotherapy, and seven underwent SCT. With a median follow-up of 2.9 years, the 5-year overall survival for the whole cohort was 43% ± 13. Five additional patients died after more than 90 days the diagnosis of *Fusarium* species infection: four died of progressive underlying malignant disease and one from other SCT-related infections.

## 4. Discussion

To the best of our knowledge, this is the largest population-based study of invasive fusariosis in children, consisting of 22 patients from all six pediatric hematology–oncology departments in Israel Our most important findings are the considerably better outcomes in children compared to those reported in adults: the 90-day survival rate for this pediatric cohort with invasive fusariosis was 77%, with 80% of deaths attributed to *Fusarium* infection. Notably, death attributed to fusariosis was encountered only in patients with relapsed/refractory hematological malignancies.

Most of the cases in our study involved children with acute leukemias (ALL and AML). In adult studies, the vast majority of cases of invasive fusariosis involved patients with either hematological malignancies and neutropenia or in SCT recipients with either neutropenia or active GVHD [23,24]. Interestingly, in our study, patients with malignant hematological disorders comprised only 73% of the study cohort compared to 90% reported for adults [24]. It was also notable that six of our 22 pediatric cases involved patients with non-malignant hematological conditions and prolonged neutropenia (4/22; 18%) or solid tumors (2/22; 9%). These pediatric solid tumors included medulloblastoma and neuroblastoma, which are both currently treated with intensive chemotherapeutic regimens and adjunctive immunotherapy. Importantly, invasive fusariosis occurred in patients with non-malignant hematological conditions, even in the absence of preceding immunosuppressive therapy.

Sixteen of our twenty-two patients (73%) presented with disseminated fusariosis, five patients (23%) with local disease (sinus or skin), and one patient with isolated fungemia. Comparable to the adult rates, 36% of our cohort had bloodstream infections. Fungemia is a common manifestation of invasive fusariosis and usually occurs in the context of disseminated disease. The prevalence of bloodstream infections is reportedly related to the production of yeast-like structures (aleurioconidia) by some *Fusarium* species, and those structures have the capacity to invade the bloodstream [4]. Isolated fungemia, in the absence of additional organ involvement, was present in one of our study patients who had an underlying solid tumor and normal neutrophil counts, and it was most likely related to a central venous catheter with no concurrent tissue invasion [20,21].

Skin involvement was the most common manifestation in our study, reported in 64% of patients, followed by sinus involvement in 41%, with both rates similar to those reported in adult studies [24]. Pulmonary involvement was notably rare (18%) compared to the significantly higher rates (50%) reported for adults [24,25]. Interestingly, clinical and radiological bone/joint involvement was reported in 4/22 patients (18%), in contrast to anecdotal reports among adults [26,27,28]. Bacterial osteoarticular infections of hematogenous origin are also reportedly more common in children than in adults owing to differences in skeletal structure and vascularization [29]. Such anatomical differences may also explain the more frequent osteoarticular involvement in childhood fusariosis.

The most striking difference between the results of our study and those reported for adults was the significantly better outcome. Ninety-day probability of survival for the whole cohort was 77%, exceedingly better than outcomes reported for adult patients. Boutati et al. reported an adult 90-day survival rate of 30% [23], Lortholary et al. reported a rate of 42% [30], Campo et al. reported a rate of 34% [31], and Nucci et al. reported an improved outcome of 43% for patients treated between the years 2001–2011, compared to 21% for patients treated between the years 1985–2000 [24]. Better outcomes in this pediatric cohort, compared to those reported in adults, may have been influenced by differences in infection patterns, such as the limited numbers of patients with pulmonary involvement. Importantly, all four of our patients who died with active fusariosis were neutropenic and died with relapsed/refractory uncontrolled acute leukemia. The ninety-day probability of survival for our patients with relapsed/refractory underlying malignancy was 33% compared to 94% for those with non-refractory disease (*p* < 0.001).

Nineteen of our patients (86%) were treated with combination antifungal therapy and twenty-one (95%) received azole therapy. There are no controlled randomized studies that evaluated the efficacy of different treatment regimens for invasive fusariosis. Most retrospective studies of invasive fusariosis have reported therapeutic strategies with combined antifungal agents, usually voriconazole with liposomal amphotericin B or other agents [30,32], with similar response rates attained by this strategy to those achieved by monotherapy [24]. An initial therapeutic strategy of combined antifungal agents is widely in use due to the high mortality rates of this condition and the frequently high minimal inhibitory concentrations for voriconazole and polyenes. No association between high MIC values and patient outcome, however, has been shown thus far, and the clinical significance of the observed in vitro high MIC values of *Fusarium* species to many antifungal agents remains largely unclear [5,33,34].

The recent European Conference on Infections in Leukemia 2020 guidelines for the treatment of invasive fungal diseases in pediatric patients with cancer or post- SCT recommend that rare fungal infections, such as fusariosis, should be treated according to the recommendations for the adult populations [35]. Current global guidelines for the diagnosis and management of rare mold infections, as developed by the European Confederation of Medical Mycology, the International Society for Human and Animal Mycology (ISHAM), and the American Society for Microbiology (ASM) [36], strongly recommend liposomal amphotericin or voriconazole for primary treatment and also endorse an initial approach of combination therapy, with potential early stepdown to monotherapy. In our study, despite the widely variable MIC values for various antifungal agents, and despite the uncertain pharmacokinetics of voriconazole in children [37], therapy achieved considerably higher survival rates than those attained in the adult population.

As for adjunctive therapy modalities, global guidelines [36] strongly recommend surgical debridement procedures, as reported herein for ten study patients. In addition, nine study patients (41%) were treated with G-CSF and seven (32%) with granulocyte transfusions. Although recovery of immunity is recognized as an important prognostic factor in invasive fusariosis, the effect of granulocyte transfusions and G-CSF administration upon fusariosis outcome has not yet been demonstrated [38].

The significance of antifungal susceptibility testing for treatment selection and outcome of patients with invasive fusariosis remains undetermined. Nonpharmacological parameters related to immune reconstitution, such as neutrophil recovery, however, were shown to be of critical significance [24,25,31,39,40]. Immune reconstitution is a key prognostic factor in the outcome of invasive fusariosis. Patients with refractory uncontrolled leukemia fail to achieve hematopoietic recovery and are unable to undergo immune reconstitution, which probably explains the high mortality rates attributed to invasive fusariosis. Thus, the higher survival rates of invasive fusariosis in the pediatric population compared to those of adults may be explained by host factors, but also by the vastly different spectrum and considerably superior outcomes of hematological malignancies in children compared to their adult counterparts [9].

The limitations of this study include its small sample size and its retrospective nature, both due to the rarity of this condition.

## 5. Conclusions

This is the largest population-based study of pediatric invasive fusariosis. The majority of patients had underlying hematological malignancies, but a significant proportion had benign hematological conditions and solid tumors. The clinical manifestations were similar to those described in the adult population, but with significant involvement of bones/joints and lower rates of pulmonary involvement. The most common clinical manifestation was skin involvement, with distinctive erythematous papular or nodular skin lesions. A high index of suspicion for fungal infections should be maintained when treating neutropenic children, even those with non-malignant hematological conditions, and skin lesions should be promptly biopsied. The ninety-day survival rates in our pediatric cohort were strikingly higher than those reported in adults, demonstrating that invasive fusariosis is a life-threatening but salvageable condition in immunosuppressed children.

## Figures and Tables

**Table 1:** Demographics, infection patterns, and treatment of patients.

Characteristics			All (*n* = 22) *n* (%)	Death ≤ 90 d (*n* = 5) *n* (%)	Alive ≤ 90 d (*n* = 17) *n* (%)	*p* Value *
Sex	Male		14 (64)	4 (80)	10 (59)	0.61
	Female		8 (36)	1 (20)	7 (41)	
Age (years)	<10		9 (41)	1 (20)	8 (47)	0.36
	≥10		13 (59)	4 (80)	9 (53)	
	Median (range)		10.8 (0.4–18)	13.2 (8.1–18)	10.5 (0.4–16)	
Underlying condition	Hematological malignancies		16 (73)	5 (100)	11 (65)	0.27
		ALL	8	4	4	
		AML	8	1	7	
	Solid tumors		2 (9)	0	2	
		Neuroblastoma	1	0	1	
		Medulloblastoma	1	0	1	
	Non-malignant hematological conditions		4 (18)	0	4	
		Aplastic anemia	2	0	2	
		Beta-thalassemia post-SCT	1	0	1	
		Congenital neutropenia due to ADA2 deficiency	1	0	1	
Disease status	Relapsed/refractory malignancy Non-refractory		6 (27) 16 (73)	4 (80) 1 (20)	2 (12) 15 (88)	0.009
Preceding corticosteroid therapy	Yes		7 (32)	3	4	0.27
	No		15 (68)	2	13	
Preceding treatment	SCT		9(41)	4 (80)	5 (29)	0.12
		Allogeneic SCT	8	4	4	
		Autologous SCT	1	0	1	
		Concurrent GVHD	0	0	0	
		Time from SCT, days Median (range)	35 (1–1174)	122 (35–1174)	8 (1–194)	
	Other	Chemotherapy	9 (41)	1 (20)	8 (47)	
		Immunotherapy	2 (9)	0	2	
		None	2 (9)	0	2	
Neutropenia	Yes	New-onset	17 (77)	4	13	1.00
		Congenital	2 (9)	0	2	
	None		3 (14)	1	2	
Blood cultures	Positive Negative		8 (36) 14 (64)	2 3	6 11	1.00
Pattern of infection	Disseminated		16 (73)	5	11	0.27
	Local		5 (23)	0	5	
	Isolated fungemia		1 (4)	0	1	
Category (EORTC/MSG criteria)	Proven		19 (82)	5	14	-
	Probable		3 (18)	0	3	
Antifungal therapy	Combination therapy		19 (86)	4	15	-
		L-AmB -azole combination ^a^	11	0	13	
		AmB-voriconazole combination	7	3	4	
		Other ^b^	1	1	0	
	Single-agent therapy		3 (14)	1	2	
Antifungal prophylaxis	Yes		19 (86)	5	14	-
		Fluconazole	10	2	8	
		Itraconazole	7	2	5	
		Voriconazole	1	0	1	
		Caspofungin	1	1	0	
	No		3 (14)	0	3	

* Fisher’s exact test. ^a^ L-AmB + voriconazole (*n* = 6); L-AmB + posaconazole (*n* = 2); L-AmB + isavuconazole (*n* = 3). ^b^ Voriconazole + caspofungin. Abbreviations: ADA = adenosine deaminase; ALL = acute lymphoblastic leukemia; AmB = amphotericin B deoxycholate; AML = acute myeloid leukemia; EORTC/MSG = European Organization for Research and Treatment of Cancer/Mycoses Study Group; G-CSF = granulocyte colony-stimulating factor; L-AmB = liposomal amphotericin B; SCT = stem cell transplantation.

**Table 2:** Microbiological data and antifungal susceptibility testing in 11 cases: minimal inhibitory concentrations (mcg/mL).

Patient No. *	Pathogen	Source	EORTC Classification	Amphotericin B	Voriconazole	Fluconazole	Itraconazole	Posaconazole	Caspofungin	Anidulafungin	Isavuconazole
1	*F. solani*	Skin, blood-cultures. BAL-PCR.	Proven	2	>16		>16	>16	>8	>16	
4	*F. solani*	Skin, blood-culture and PCR	Proven	2	3						
6	*Fusarium* sp.	Skin, sinus, blood-cultures	Proven	2	2			2			
7	*Fusarium* sp.	Skin culture	Proven	0.5	>16		>16	>16	>8	>16	>16
8	*Fusarium* sp.	Sinus culture	Probable	2	>16		>16	>16	>8	>8	
15	*Fusarium* sp.	Blood culture, and histopathology from skin lesion	Proven	24	0.75	192	32	0.38			
17	*F. moniliforme*	Culture and PCR from sinus, histopathology from skin lesions	Proven	16	0.75						
18	*F. solani*	Culture, histopathology and PCR from sinus	Proven	1.5	0.75	256	32				
20	*Fusarium* sp.	Culture, histopathology and PCR from sinus	Proven	4	4	128	16	>8			
21	*Fusarium* sp.	Blood culture	Proven	4	0.47						
22	*Fusarium* sp.	Culture and histopathology from sinus	Proven	2	4	128	16	2	>8	>8	

BAL, bronchoalveolar lavage; PCR, polymerase chain reaction. * Detailed data for all study patients are summarized in Supplementary Table S1.

## Data Availability

Deidentified data presented in this study are available on request from the corresponding author.

## Supplementary Materials

The following supporting information can be downloaded at: https://www.mdpi.com/article/10.3390/jof8040387/s1, Table S1: Patient Demographics, Treatment and Microbiological Data.
