# Supplementary material for: Invasive Fusariosis in Pediatric Hematology/Oncology and Stem Cell Transplant Patients: A Report from the Israeli Society of Pediatric Hematology-Oncology
Source: J Fungi (Basel). 2022 Apr 11;8(4):387. doi: 10.3390/jof8040387 (PMC9030963; doi:10.3390/jof8040387)
Supplement: Supplementary file 1 [file jof-08-00387-s001.zip › jof-1625624-SI.pdf]

Supplementary Table S1: Patient Demographics, Treatment and Microbiological Data

Patient no.	Sex/ age (yrs)	Underlying disorder	Preceding treatment	Prior SCT? (1 yr)	Corticosteroid pre-treatment ?	Duration of neutropenia (days)	Antifungal prophylaxis	Pathogen	Sites of involvement	Diagnostic methods	EORTC/ MSG category	Antifungal therapy	90-day outcome	Adjunctive therapy
1	F/0.4	AML	Chemotherapy (induction)	No	No	30	Voriconazole	*F. solani*	Skin, blood, bone/joints, lungs	Cultures and PCR from blood, bronchoalveolar lavage and skin.	Proven	L-AmB + posaconazole	alive	Surgery
2	F/13	Relapsed ALL	Chemotherapy (consolidation)	No	Yes	16	Fluconazole	*F. dimerum*	Skin, liver, spleen, thyroid	Skin culture and PCR	Proven	L-AmB + voriconazole	alive	Granulocyte infusions<br><br>GCSF
3	M/1.1	Aplastic anemia	Immunotherapy (ATG/CSA)	No	No	Congenital	Fluconazole	*F. proliferatum*	Skin, bone/joints	Skin culture and PCR	Proven	L-AmB + voriconazole	alive	Granulocyte infusions
4	M/13.8	AML	SCT	Yes	No	11	Fluconazole	*F. solani*	Skin/blood	Blood culture and PCR	Proven	L-AmB + voriconazole	alive	-
5	M/13.2	Relapsed ALL	SCT	Yes	Yes	54	Fluconazole		Skin, blood	Blood culture	Proven	Voriconazole + caspofungin	Death with active fusarium	-
6	F/14	Thalassemia	SCT	Yes	No	17	Fluconazole		Skin, sinus, blood	Cultures from skin, sinus and blood	Proven	AmB + voriconazole	alive	Granulocyte infusions
7	F/10.5	Relapsed ALL	SCT	Yes	No	60	Fluconazole		Skin	Culture from skin lesion	Proven	L-AmB + isavuconazole	alive	GCSF

8	M/3.8	Congenital neutropenia due to ADA2 deficiency	None	No	No	Congenital	Fluconazole		Sinus	Culture from sinus	Probable	L-AmB + isavuconazole	alive	Granulocyte infusions Surgery
9	M/16	Aplastic anemia	None	No	No	30	Fluconazole		Sinus	Culture from sinus	Probable	L-Amb + isavuconazole	alive	Granulocyte infusions Surgery
10	M/8.1	Relapsed ALL	SCT	Yes	No	60	Fluconazole		Skin, sinus	Cultures from skin and sinus	Proven	AmB + voriconazole	Death with active fusarium	Granulocyte infusions
11	M/14	AML	Chemotherapy (consolidation)	No	No	11	Itraconazole		Skin, lung, liver	Skin PCR and histopathology	Proven	L-AmB + voriconazole	alive	GSCF
12	M/18	Relapsed ALL	Chemotherapy	Yes	Yes	6	Itraconazole		Sinus, lung	Cultures and histopathology from sinus	Proven	AmB	Death with active fusarium	Granulocyte infusions GCSF Surgery
13	M/13	AML	Chemotherapy (consolidation)	No	No	10	Fluconazole		Skin, bone/joints/eyes	Cultures from synovial fluid	Proven	AmB + voriconazole	Alive	-
14	M/3	ALL	Chemotherapy (induction)	No	Yes	50	None		Skin (disseminated)	Skin culture	Proven	AmB + voriconazole	Alive	GCSF
15	M/11	AML (Fanconi)	SCT	Yes	No	105	Itraconazole		Skin, bone, blood	Blood culture, and histopathology from skin lesion	Proven	L-AmB + posaconazole + terbinafine	alive	GSCF
16	M/10.3	Relapsed ALL	Chemotherapy (induction)	No	Yes	14	Itraconazole	*F. solani*	Skin, blood	Blood culture, PCR from skin lesion	Proven	AmB + voriconazole	Death with active	-

													fusarium	
17	F/15.8	AML	SCT	Yes	No	No	Caspofungin	*F. moniliforme*	Skin, sinus	Culture and PCR from sinus, histopathology from skin lesions	Proven	AmB + voriconazole	Death not attributed to fusarium	Surgery
18	F/16	AML	Chemotherapy (consolidation)	No	No	7	Itraconazole	*F. solani*	Skin, sinus	Culture, histopathology and PCR from sinus	Proven	AmB + voriconazole	alive	Surgery GCSF
19	F/6	Neuroblastoma	Immunotherapy (anti GD2)	Yes (autologous)	No	No	None	*F. solani*	Blood, lungs	Blood culture and PCR	Proven	Voriconazole	alive	
20	F/2	AML	Chemotherapy (induction)	No	No	30	Itraconazole		Sinus	Culture, histopathology and PCR from sinus	Proven	L-AmB + voriconazole	alive	Surgery GCSF
21	M/5.3	Medulloblastoma	Chemotherapy	No	Yes	No	None		Blood only	Blood culture	Proven	Voriconazole	alive	
22	M/4.2	ALL	Chemotherapy (consolidation)	No	Yes	17	Itraconazole		Sinus	Culture and histopathology from sinus	Proven	L-AmB + voriconazole	alive	Surgery GCSF

ADA= adenosine deaminase; ALL=acute lymphoblastic leukemia; AmB=amphotericin B; AML=acute myeloid leukemia; EORTC/MSG= European Organization for Research and Treatment of Cancer/Mycoses Study Group; G-CSF= granulocyte colony-stimulating factor; L-AmB=liposomal amphotericin B; SCT=stem cell transplantation
